# Chronic non-specific low back pain influences mechanics during walking and transferring load among nurses

**DOI:** 10.1080/07853890.2025.2525402

**Published:** 2025-06-30

**Authors:** Nur Athirah Abd Rahman, Mohd Imran Yusof, Shazlin Shaharudin

**Affiliations:** ^a^ Defence Fitness Academy, Universiti Pertahanan Nasional Malaysia, Kuala Lumpur, Malaysia; ^b^ Exercise and Sports Science Programme, School of Health Sciences, Universiti Sains Malaysia, Kota Bharu, Kelantan, Malaysia; ^c^ Department of Orthopedics, School of Medical Sciences, Universiti Sains Malaysia, Kota Bharu, Kelantan, Malaysia

**Keywords:** Human health, kinematics, kinetics, motion, spine

## Abstract

**Background:**

This study aimed to compare full-body biomechanical outcomes, during walking and 180° lateral transfer between healthy and chronic non-specific low back pain (CNLBP) nurses.

**Methods:**

A total of 26 female nurses (healthy group = 13 and CNLBP group = 13) with at least 2 years of work experience were recruited. Three-dimensional (3D) motion analyses of full-body kinematics and kinetics were captured during walking and 180° lateral load transfer. An independent *t* test was used to compare the differences across groups.

**Results:**

There were significant differences in biomechanical factors in the frontal plane during walking and 180° lateral transfer. The results indicate that those with CNLBP walk with more lumbar, hip, knee and ankle adduction, and lateral pelvic tilt at initial contact and maximum vertical ground reaction force (VGRF) in dominant compared to the healthy group. Moreover, those with CNLBP lifted the load with more lumbar and ankle abduction and less hip and knee abduction then dropped the load with more lumbar abduction and less hip and knee abduction with lateral pelvic tilt than the healthy group.

**Conclusions:**

Nurses with CNLBP employed greater lumbar flexion during walking and greater abduction during 180° lateral transfer than healthy nurses. This motion caused the upper body to move toward the load and decreased the VGRF, thereby indirectly applying the narrow base transfer technique. Furthermore, an increase in lumbar flexion during walking among nurses with CNLBP was common, as a compensatory mechanism to avoid pain.

## Introduction

Low back pain (LBP) is a leading musculoskeletal disorder that affects all populations worldwide [1]. Those with LBP experience pain and discomfort above the inferior gluteal folds, below the costal margin, and with or without referred leg pain [2]. There are three subtypes of LBP based on the duration of pain namely acute (LBP lasted for less than 6 weeks), sub-acute (LBP lasted between 6 weeks and 3 months), and chronic (LBP persisted for longer than 3 months or recurrent LBP) [2–4]. Furthermore, LBP can be classified as either specific or non-specific type based on their underlying pathological mechanism [5]. The non-specific LBP can be further divided into mechanical or non-mechanical LBP. Mechanical LBP refers to back pain that is caused or aggravated by movements, postures, or mechanical stress on the spine, such as during bending, lifting, or twisting. It generally involves the muscles, ligaments, or joints of the spine and is characterized by pain that often worsens with movement and improves with rest [5]. Non-mechanical LBP usually not relieved by rest, often caused by underlying systemic, inflammatory, or pathological conditions that affect the spine and surrounding tissues, that can include infections, tumors or inflammatory conditions [5]. This study focused on LBP persisting for longer than 3 months accompanied with non-specific mechanical LBP causes and symptoms.

Compared to other healthcare workers, nurse is a high-risk profession associated with LBP. Nurses’ job scope involves lifting and moving patients from chairs or beds, helping patients ambulate, moving the bed, frequent standing, awkward postures consist of deep bending and twisting of the trunk during patient-handling tasks, and forceful exertion while handling obese or overweight patients, which lead to the development of LBP [6]. Their daily life activities, such as psychological problems, interpersonal relations, and quality of life, can be affected by LBP [1]. Furthermore, they may be forced to quit their jobs or change their workplace because of LBP [7]. Nurses who work in shifts, particularly the morning shift, reported a higher probability of LBP than nurses who work in clinical hours [8]. In addition, the total years of nursing experience and working experience in the current ward were associated with LBP among nurses [9]. These two factors explain why nurses with more than 20 years of working experience contributed the highest number of LBP cases compared to nurses working less than one year [9]. Prevalence statistics of LBP among nurses were 87.5% in Sudan, 65% in Nepal, 72% in Taiwan, and 50–80% worldwide with about 67% to 84.2% of them were nurses from intensive care units [10]. Despite high prevalence of LBP in nurses, biomechanical studies on full-body kinematics and kinetics among nurses have not yet been reported, as previous studies have focused on qualitative outcomes such as job satisfaction [11] and psychosocial factors associated with LBP [12]. Detailed mechanics among nurses with CNLBP will provide the insight of clinical practice to differentiate the abnormal versus normal movement particularly during performing tasks related to their job [13]. This gap contributes to the limited understanding of why nurses experience a high prevalence of LBP. Besides that, due to wide variation of LBP causes and symptoms, we selected a homogenous group of participants (females nurses with chronic non-specific LBP (CNLBP)) and conducted this study especially in the frontal plane during walking and carrying and transferring a load. Hence, the current study aimed to compare the full-body mechanics during walking and carrying and transferring a load between healthy female nurses and those with CNLBP.

## Materials and methods

### Study design and participants

A cross-sectional study design with a purposive sampling method was used to recruit female nurses with at least 2 years of working experience from a local hospital between October 2022 and November 2022. The inclusion criteria for the CNLBP group were diagnosed with CNLBP persisting for longer than 3 months, history of LBP relapses, with at least two painful episodes per year, each with an intensity greater than five on the Visual Analog Scale (VAS), the worst episode of LBP occurred within 2 years prior to testing and required conservative treatment, absence of ‘red flags’ indicating specific causes of LBP, such as inflammatory disease or cauda equina syndrome, absence of dominant ‘yellow flags,’ including behaviours, emotions, or beliefs that may influence pain experience, pain-free status at the time of testing and no cardiovascular diseases.

For both groups, participants were excluded if they were diagnosed with spinal deformity, being pregnant or 6 months postpartum at the time of the study, had other serious medical conditions, had a history of previous spine surgery, history of a spinal fracture, history of neurologic deficits, inflammatory joint disease, rheumatic disease, or a history of chronic pain syndrome or other serious medical conditions. All the participants provided written informed consent. The study protocol was approved by the Human Research Ethics Committee Universiti Sains Malaysia (USM/JEPeM/19100637) and conducted according to the Declaration of Helsinki.

There was no randomisation for both groups as participants were assigned according to the presence of CNLBP diagnosis. The outcome assessor was blinded to the participants’ group to ensure no outcome bias.

### Experimental protocol

Initially, an orthopaedic spine physician performed the assessment and classified the participants into specific groups (i.e. healthy and CNLBP). Participants’ body weight (kg), standing height (cm), leg length (cm), and body mass index (BMI) (kg/m^2^) were measured. Next, they warmed up for 5 min. Then, their three-dimensional (3D) full-body kinematics and kinetics were evaluated during walking and transferring a standard load by using a 3D Motion Capture System (six infrared cameras, Qualisys Track Manager, version 2.6.673, Gothenburg, Sweden). Forty-six reflective markers (25-mm diameter) were placed on both sides of the head, glabella, jugular notch, xiphoid process of the sternum, C7 spinous process, T2 spinous process, midpoint between inferior angles of most caudal points of the scapulae, L1, L3, and L5 spinous process, both sides of the acromion, lateral epicondyle of the humerus and elbow, right wrist (1st finger and 5th finger), left wrist (1st finger and 5th finger), both sides of the forefinger (2nd finger), anterior superior iliac spines, posterior superior iliac spines, greater trochanter (1/3 from proximal end), lateral knee, medial knee, proximal tip of the head of the fibula, middle of tibial, lateral malleolus, medial malleolus, metatarsal head (1st, 2nd and 5th finger), and posterior heel. Marker placement was performed following the standard IOR Gait method [14].

This study involved two evaluation sessions (3D motion capture and clinical tasks), with each session lasted approximately 2 h. First, the participants walked at their self-selected speed across a 3 m walkway. A successful trial was defined as a trial in which the participants walked naturally with the whole foot of the leading leg landed on the force platform (Bertec, Columbus, Ohio, USA).

The carrying and transferring a standard load [15] involved the participant performing a 180° lateral load transfer using a box weighing 5% of their body weight. They were required to lift the box rather than slide it off the surface, determine their own maximum transfer distance without lifting the foot completely off the ground, and drag or slide the feet. Moreover, they must place all fingers on the box handles before lifting the box, lean with the body weight against the box, and ensure that the box is completely on the pedestal without pushing against the box.

During these trials, the trajectories of the reflective markers were sampled at 100 Hz and identified using Qualisys Track Manager software. Then, the raw data of the marker coordinates were low-pass filtered using a fourth-order zero-lag Butterworth filter with a cutoff frequency of 12 Hz. Missing trajectories were pattern-filled using spline estimates. Next, the data were transferred to Visual 3D (version 5, C-Motion Inc., Rockville, MD, USA) to construct a bone model and calculate the kinematic and kinetic variables of the lumbar, pelvic, hip, knee, and ankle joints. The kinematic and kinetic data from these two trials (i.e. walking, carrying, and transferring a standard load) were analysed during the initial contact (IC) phase and maximum vertical ground reaction force (VGRF) phase. IC was defined as the point in the trial when the vertical GRF exceeded 10 N [16] whereas maximum VGRF phase was defined as magnitude and pattern of maximum mechanical loading at the foot in the vertical direction [17].

### Statistical analyses

Data analysis was performed using IBM SPSS 27.0. Descriptive statistical analysis was conducted to determine the mean, standard deviation (SD), and range of physical characteristics, as well as lower extremity and lumbar kinematics during walking and load transfer. The analysis was performed using Qualisys Track Manager software and v3D modelling software. All data were tested for normal distribution using the Shapiro–Wilk test. An independent *t* test was conducted to compare the mechanical data during selected motions, and anthropometric data between the two groups. The level of significance was set at *p* value <0.05.

## Results

### Physical characteristics of participants

All participants successfully completed the experimental procedure. Healthy group had 10.38 ± 4.05 year of working experience as a staff nurse whereas CNLBP group had 14.46 ± 7.46 year of working experience in the same field. Table 1 presents the physical characteristics of each group. There were no significant differences in age, weight, or body mass index (BMI). However, the results showed that the participants in the healthy group were taller with longer dominant and non-dominant leg lengths measured in supine and standing positions than those in the CNLBP group.

**Table 1.** Physical characteristics of participants.

	Healthy group (*n* = 13)	CNLBP group (*n* = 13)	*p* value
Age (years)	36.00 (5.05)	36.77 (6.67)	0.71
Height (cm)	157.89 (3.17)*	153.60 (3.76)	0.03
BMI (kg/m^2^)	21.47 (2.04)	20.77 (1.27)	0.40
Weight (kg)	53.73 (7.22)	49.14 (4.83)	0.16
DLSU length (cm)	88.61 (1.82)*	83.71 (3.35)*	0.01
DLST (cm)	87.93 (1.98)*	82.95 (3.01)*	0.01
NDLSU (cm)	87.94 (2.05)*	83.08 (3.21)*	0.03
NDLST (cm)	87.55 (2.07)*	82.62 (3.33)*	0.02
Dominant (right)	69.23%	100%	–

CNLBP chronic non-specific low back pain; DLSU dominant leg length in supine position; DLST dominant leg length in standing position; NDLSU non-dominant leg length in supine position; NDLST non-dominant leg length in standing position; BMI body mass index.

Values are presented as mean (standard deviation).

*Significant difference between the two groups. All presented data are original and derived from the experimental procedures.

### Joint angle in frontal plane during walking

Table 2 presents the kinematics of the dominant leg in the frontal plane during walking. The results indicate that those with CNLBP walk with more lumbar, hip, knee, and ankle adduction and lateral pelvic tilt at IC and maximum VGRF in the dominant leg compared to the healthy group.

**Table 2.** Joint angle of dominant leg during walking at initial contact and maximum VGRF in frontal plane across healthy and CNLBP group.

Phases/joint angle (°)	Healthy (*n* = 13)	CNLBP (*n* = 13)	*p* value
Initial contact			
Lumbar angle (°)	3.30 (0.23)*	11.20 (2.39)*	0.003
Pelvic angle (°)	−2.45 (0.97)*	−6.81 (4.71)*	0.028
Hip angle (°)	20.56 (2.05)*	24.89 (6.14)*	0.024
Knee angle (°)	3.20 (0.72)*	7.70 (1.10)*	0.001
Ankle angle (°)	3.37 (0.48)*	7.05 (0.56)*	0.006
Maximum VGRF			
Lumbar angle (°)	3.29 (0.34)*	10.77 (2.08)*	0.009
Pelvic angle (°)	−3.95 (0.82)*	−6.95 (1.74)*	0.005
Hip angle (°)	19.95 (1.97)	22.98 (7.13)	0.153
Knee angle (°)	5.80 (1.46)	5.64 (4.30)	0.904
Ankle angle (°)	3.18 (0.57)*	4.72 (1.23)*	0.001

CNLBP chronic non-specific low back pain; VGRF vertical ground reaction force.

Values are presented as mean (standard deviation).

*Significant difference between the two groups.

^+^Values indicate adduction of the lumbar, pelvic, hip, knee, and ankle joints. All presented data are original and derived from the experimental procedures.

### Kinetics in frontal plane during walking

Table 3 lists the force, moment, and centre of pressure (COP) of the dominant leg in the frontal plane during walking for each group. There were no significant differences in force, moment, and COP of the dominant leg during walking at IC and maximum VGRF in the frontal plane between the healthy and CNLBP groups.

**Table 3.** Force, moment and centre of pressure (COP) of full body at dominant leg during walking at initial contact and maximum VGRF in frontal plane across healthy and CNLBP group.

Phases/kinetics variable	Healthy (*n* = 13)	CNLBP (*n* = 13)	*p* value
Initial contact			
Force (N)	37.66 (31.66)	41.92 (12.88)	0.657
Moment (Nm)	1.64 (0.66)	2.49 (2.34)	0.222
COP (Nm^–2^)	−15.69 (183.14)	−145.6 (304.08)	0.199
Maximum VGRF			
Force (N)	503.58 (69.67)	537.04 (35.30)	0.136
Moment (Nm)	25.34 (10.52)	29.59 (19.91)	0.503
COP (Nm^–2^)	16.76 (118.96)	17.28 (40.73)	0.988

CNLBP chronic non-specific low back pain; VGRF vertical ground reaction force; COP center of pressure.

Values are presented as mean (standard deviation).

*Significant difference between the two groups.

^+^Values indicate adduction of the lumbar, pelvic, hip, knee, and ankle joints. All presented data are original and derived from the experimental procedures.

### Joint angle in frontal plane during carrying and transferring a standard load

Table 4 shows the kinematics of the ipsilateral leg in the frontal plane while carrying and transferring a standard load. Those with CNLBP lifted the load with more lumbar and ankle abduction and less hip and knee abduction and then dropped the load with more lumbar abduction and less hip and knee abduction with lateral pelvic tilt than the healthy group.

**Table 4.** Joint angle of ipsilateral leg during carrying and transferring a standard load at start and end in frontal plane across healthy and CNLBP group.

Phases/joint angle (°)	Healthy (*n* = 13)	CNLBP (*n* = 13)	*p* value
Start			
Lumbar angle (°)	4.48 (0.39)*	20.55 (0.58*	0.003
Pelvic angle (°)	60.66 (4.19)	59.65 (12.72)	0.789
Hip angle (°)	25.71 (0.83)*	9.26 (0.49)*	0.008
Knee angle (°)	3.61 (0.52)*	1.95 (1.58)*	0.001
Ankle angle (°)	3.63 (0.34)*	4.02 (0.44)*	0.019
End			
Lumbar angle (°)	7.53 (0.37)*	10.67 (1.86)*	0.017
Pelvic angle (°)	69.17 (0.77)*	65.00 (5.18)*	0.008
Hip angle (°)	30.28 (2.74)*	21.33 (3.33)*	0.021
Knee angle (°)	3.48 (0.21)*	2.55 (0.21)*	0.033
Ankle angle (°)	4.44 (0.31)	4.19 (0.45)	0.112

CNLBP chronic non-specific low back pain.

Values are presented as mean (standard deviation).

*Significant difference between the two groups.

^+^Values indicate abduction of the lumbar, pelvic, hip, knee, and ankle joints. All presented data are original and derived from the experimental procedures.

### Kinetics in frontal plane during carrying and transferring a standard load

Table 5 shows the kinetics of the ipsilateral leg in the frontal plane while carrying and transferring a standard load. Those with CNLBP dropped the load with a greater moment and greater COP exerted on the body than the healthy group.

**Table 5.** Force, moment and centre of pressure (COP) of full body at the ipsilateral leg during carrying and transferring a standard load at start and end in frontal plane across healthy and CNLBP group.

Phases/kinetics variable	Healthy (*n* = 13)	CNLBP (*n* = 13)	*p* value
Start			
Force (N)	357.00 (84.34)	390.02 (91.07)	0.347
Moment (Nm)	86.61 (22.05)	99.04 (39.21)	0.329
COP (Nm^–2^)	−75.06 (72.13)	−89.04 (9.69)	0.495
End			
Force (N)	2.10 (148.81)	53.48 (24.94)	0.231
Moment (Nm)	14.63 (5.52)*	32.45 (26.09)*	0.024
COP (Nm^–2^)	−63.92 (101.06)*	−121.69 (18.59)*	0.054

CNLBP chronic non-specific low back pain; VGRF vertical ground reaction force; COP center of pressure.

Values are presented as mean (standard deviation).

*Significant difference between the two groups.

^+^Values indicate abduction of the lumbar, pelvic, hip, knee, and ankle joints. All presented data are original and derived from the experimental procedures.

## Discussion

### Physical characteristics of participants

The mean value of BMI for both healthy and CNLB groups are 21.47 kg/m^2^ and 20.77 kg/m^2^, respectively (Table 1). This finding shows that all participants had a healthy weight and a normal BMI [18]. Additionally, structural and postural changes in the CNLBP group resulted in a shorter leg length in both standing and supine positions compared to the healthy group. Although the cause of LBP due to leg length remains unclear, nursing related tasks may require them to reach farther especially during transferring a patient. Over time, this may cause pain among those who are short [19].

### Joint angle in frontal plane during walking

Similar to our findings (Table 2), those with CNLBP typically exhibit lumbar adduction at IC and maximum VGRF during walking [20]. In the frontal plane, the results (Table 2) showed that those with CNLBP walked with knee valgus (i.e. more knee adduction) and excessive foot pronation (i.e. more ankle adduction), as also observed in a previous study [21]. Those with LBP altered their walking pattern to have a more stable walking motion, which acts as a protective strategy to prevent painful motion and further injuries [21]. However, they will develop pain and injury in the long-term effects if the correct walking posture is not practiced [21].

### Kinetics in frontal plane during walking

The results (Table 3) showed more force acting toward the body during IC and maximum VGRF in CNLBP group than in healthy group. This is because those with CNLBP walked with excessive pronation (more ankle adduction) [22]. The LBP group with pronated foot exerted greater force (*p* = 0.001), whereas the healthy group with normal foot exerted less force than the healthy group with pronated foot (*p* < 0.05) [22]. Lower limb mechanical abnormalities were associated with lower back segments, which may lead to LBP patients to experience higher forces and greater loading rates compared to controls [22]. Increased VGRF during walking occurred because of more antagonistic moments were required by the back muscles to stabilize the upper body [23]. The current study found that the CNLBP group had greater moment and more posterolateral COP of the dominant leg at IC when compared to the healthy group (Table 3). The role of the COP is to assess body sway by measuring its location [24]. Previous studies [22,24] found that patients with CNLBP exhibited significantly increased COP sway and walked with greater moments than healthy controls. These results suggest that excessive moment may increase the risk of LBP among those with pronated feet [22].

### Joint angle in frontal plane during carrying and transferring a standard load

The results (Table 4) indicate that those with CNLBP dropped the load with more abducted lumbar, adducted hip, adducted knee and lateral pelvic tilt than the healthy group. A previous study [25] compared manual transfer and two types of mechanized devices: vertical lifting and lateral transfer and repositioning devices. Based on the outcomes, they noted less pushing or pulling effort (hand forces), less spinal load and compression, and less trunk flexion when using a vertical lifting device during surgical procedure. However, this non-powered repositioning aid is time-consuming, and nurses prefer to use manual transfers rather than mechanized devices [25].

### Kinetics in frontal plane during carrying and transferring a standard load

Those in the CNLBP group lifted the load with greater moment and then dropped it with greater force, moment, and anteromedial COP exerted on the body than the healthy group (Table 5). In a 3D analysis of transferring a patient from lying to sitting at the edge of the bed, anteroposterior COP displacement was reported due to increased trunk flexion [26]. This is because an increase in trunk flexion may increase compression force at the spine [25]. Ankle plantar flexion is required to maintain the centre of gravity at the base of support during hip and knee flexion [15]. In addition, CNLBP patients applied the narrowed base-lifting technique and pivoted their feet toward the load direction [15]. This narrowed base-lifting technique potentially aim to maintain body balance during load transfer [15].

This study had a few limitations. The number of male nurses in the local hospital are limited hence, only female nurses were recruited in this study. Participants with CNLBP walked with a pronated foot. Therefore, future studies should explore the bottom-up mechanism of LBP and its potential to alleviate pain. Future studies should also expand the range of occupational sectors among patients to identify which job roles significantly contribute to the recurrence of LBP.

## Conclusion

LBP can arise from a combination of multiple factors. These include sex, age, nursing shifts, years of work experience, frequency of lifting patients from beds, poor body mechanics during lifting, and carrying heavy objects. Notably, nurses with CNLBP walked with greater lumbar adduction, forward pelvic tilt, knee valgus, excessive foot pronation, increased force exerted on the body, higher moments, and greater body sway than healthy nurses. In addition, during 180° lateral transfer, nurses with CNLBP lifted and dropped the load with more abducted joints and applied the narrowed base-lifting technique as a compensatory movement. Nurses are advised to maintain a straight back while walking, lifting, and lowering loads with an erect posture and keep the load close to their body. By following these practices, they can minimize LBP and reduce the risk of its recurrence.

## Data Availability

The data sets generated and analysed during the current study are not publicly available due to individual privacy could be compromised, but are available from the corresponding author on reasonable request.
